# Current Condition of Pannonic Salt Steppes at Their Distribution Limit: What Do Indicator Species Reveal about Habitat Quality?

**DOI:** 10.3390/plants10030530

**Published:** 2021-03-11

**Authors:** Zuzana Dítě, Róbert Šuvada, Tibor Tóth, Pavol Eliáš Jun, Vladimír Píš, Daniel Dítě

**Affiliations:** 1 Institute of Botany, Plant Science and Biodiversity Center, Slovak Academy of Sciences, Dúbravská cesta 9, 845 23 Bratislava, Slovakia; daniel.dite@savba.sk; 2 State Nature Conservancy of the Slovak Republic, Administration of the Slovenský kras National Park, Hámosiho 188, 049 51 Brzotín, Slovakia; robert.suvada@sopsr.sk; 3 Research Institute for Soil Science and Agricultural Chemistry, Centre for Agricultural Research, Herman O. út 15, 1022 Budapest, Hungary; tibor@rissac.hu; 4 Department of Environment and Biology, Slovak University of Agriculture, Tr. A. Hlinku 2, 949 76 Nitra, Slovakia; pavol.elias.jun@gmail.com; 5 Soil Science and Conservation Research Institute, Gagarinova 10, 827 13 Bratislava, Slovakia; vladimir.pis@nppc.sk

**Keywords:** alkali soils, electrical conductivity, extreme edaphic conditions, habitat naturalness, indicator species, organic carbon, total nitrogen, sodium adsorption ratio

## Abstract

Little is known about the suite of ecological conditions under which characteristic species may continue to develop under the pressure of recent habitat deterioration. We aimed to determine the niche of three indicator species of the priority habitat Pannonic salt steppes and to find out how their vegetation composition, land use, and soil chemistry mirror the current condition of their typical habitat. A plot-based vegetation survey was conducted in degraded and in pristine (reference) inland salt steppes in East-Central Europe. We confirmed decreased habitat quality at their northern geographical limit. Most of the sites there showed a strong prevalence of generalists (e.g., *Elytrigia repens*) and lack of specialists, both resulting from lowered habitat extremity and inappropriate land use (abandonment). A small proportion of plots (19%) were in the same good condition as the reference vegetation in the central area. Soil analyses revealed that the studied halophytes are able to persist on desalinized soils if the land use is suitable. The occurrence of the annual *Camphorosma annua* (*Amaranthaceae*) was driven largely by abiotic stress; grazing alone is insufficient for its long-term persistence, while the perennial *Artemisia santonicum* (*Asteraceae*) and *Tripolium pannonicum* (*Asteraceae*) have higher survival chances as they are able to coexist with generalists. Overall habitat quality can be reliably determined from the analyzed ecological conditions of indicator species. The outcomes of the presented work are relevant for conservation practice and can serve as a quick tool for assessing the current stage of other grassland habitats.

## 1. Introduction

Natural habitats have recently been subjected to species turnover due to negative vegetation changes when the original, highly specialized species pool has been substituted by generalists with a lower naturalness value [1]. Non-forest habitats with extreme edaphic conditions suitable for specialists are disappearing [2]. Halophytic vegetation also belongs to a group of highly threatened habitats hosting endangered plant and animal species [3]. They develop on salt-affected soils in large areas with a semiarid-subhumid climate [4], in Europe for instance on the East European Plain and the Iberian Peninsula [5]. In temperate zones, inland saline habitats occur as discrete islands, for example, in the North German Plain [6], while their main center of distribution is the Pannonian Basin [7].

Whether inland saline habitats have natural origins or owe their existence to the salt industry [8] or secondary salinization [9], their flora has a high representation of specialists (halophytes). They are well-adapted to such permanent abiotic stresses as soil salinity and an unstable water regime [10] and are excellent indicators of soil salinity [11,12,13]. Low species richness and high vegetation variability of saline habitats is governed by the salt content and moisture of the soil [14] which are key factors determining wetland and steppic saline habitats. The wetland type which prevails in temperate Europe includes salt springs, salt marshes, and shallow salt lake beds, which according to the European Red List of habitats [15] are classified as E6.3 Temperate inland salt marsh. The second, recognized as E6.2 Continental inland salt steppe, are alkali habitats of the class *Festuco-Puccinellietea* [16] where Pannonic salt steppes and salt marshes belong. Playing an important role in nature conservation policy, they are included as a priority habitat in Annex 1 of the Habitats Directive [17]. The habitat code within the Natura 2000 network is 1530. They occur exclusively in the Pannonian Basin in East-Central Europe [7].

Pannonic salt steppes all over their natural area are threatened [18]. In their central distribution (Hungary), in spite of the past water regulations and improper land use they were evaluated as of high naturalness [19]. On the edge of their area their conservation status was not evaluated. Habitat preferences of several representatives of Pannonic salt steppes have been published in local studies which focused on single species (*e.g., Camphorosma annua* in Janjatovič et al. [20] and *Puccinellia distans* agg. in Dajić Stevanović et al. [21]), usually in their typical (optimal) living conditions. The ecological range limit of these halophytes has not been evaluated, therefore we aimed to fill this gap. To do this, we applied a coenological approach, which along with the abiotic conditions (soil chemistry) defines a species matrix in which the target plant is able to grow beyond its optimal environment, in marginal ecological conditions. Studies with this approach are lacking, as vegetation surveys describe only typical species composition under optimal coeno-ecological conditions [22,23,24,25], not taking into account the recent, often severe vegetation changes. Determining the niche of target species provides better knowledge on the long-term survival chance of threatened halophytes. For this purpose, we selected three flagship species of Pannonic salt steppes: *Artemisia santonicum* subsp. *patens* K. M. Perss (*Asteraceae*)*, Camphorosa annua* Pall. (*Amaranthaceae*), and *Tripolium pannonicum* (Jacq.) Dobrocz. subsp. *pannonicum* (*Asteraceae*). They are potentially used as model species in conservation ecology to define their range limit from the aspect of vegetation composition, land use, and soil properties in a changing/degrading environment. We had the following aims:

(i) to analyze the ecological range limits of indicator plants of Pannonic salt steppes on their border of distribution,
(ii) to compare the current condition, threatening factors, and conservation measures of their target habitat with reference sites in their central distribution.

These specific aims outline the general idea of our study which is to find out what the ecological circumstances of indicator species reveal about the current condition of its typical habitat.

## 2. Materials and Methods

### 2.1. Study Area

**Figure 1:**
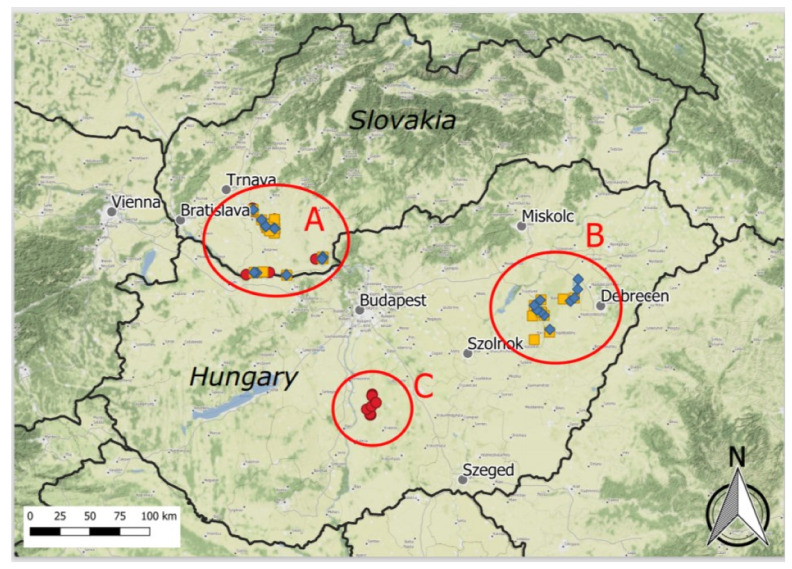
Map of the study area (Pannonian Basin) with three core areas of target habitat (Pannonic salt steppes): A—Podunajská nížina lowland—vegetation plots in several stages of habitat degradation, B—Hortobágy, and C—Kiskunság—vegetation plots (reference) in preserved salt steppes. Vegetation plots have three different symbols, one for each indicator species of Pannonic salt steppes: Blue diamond—*Camphorosa annua*, yellow square—*Artemisia santonicum* subsp. *patens*, and red circle—*Tripolium pannonicum* subsp. *pannonicum*.

The study area is situated in the Pannonian Basin, in a biogeographically discrete unit, the Pannonian bioregion [26], where three core areas of salt steppes were selected (Figure 1).

A. Podunajská nížina lowland—located in southwestern Slovakia, it is the northern enclave of Pannonic inland salt steppes with typical stands of the alliances *Puccinellion limosae* and *Festucion pseudovinae* [27], however, with the largest habitat loss and vegetation degradation. In the 1960s, the total area was estimated to be 8300 ha; recently, only 500 ha was reported [28]. The groundwater level is 1–5 m http://apl.geology.sk/gibges/accessed (accessed on 1 March 2021), and it has decreased throughout the area due to former water regulations resulting also in alteration of rare Solonchak soils to the Solonetz type. Land reclamations and abandonment beginning in the Communist era resulted in fragmentation of the habitat 1530 [29]. Only scattered locations remained in the prevailing agricultural landscape, and each has been declared as a protected area. Moderate grazing (cattle, grey cattle, and sheep) was re-established in most locations just recently.

B. Hortobágy—located in eastern Hungary as part of the Great Hungarian Plain. Meadow Solonetz soils are developed over loess with a salt accumulation zone in deeper soil layers [30]. Groundwater is rich in sodium bicarbonate (NaHCO_3_), whereas the groundwater table is relatively high (usually 0.5–2.5 m). The prevailing vegetation types are alkali steppes of *Festucion pseudovinae* which have dominated the area since the late Pleistocene. Although river regulations in the second half of the 19th century decreased regular flooding and drainage works during the 20th century dried out the marshy depressions [7], the area is now a national park harboring diverse sodic steppes with high degree of continuality. They are known as one of the best-preserved grassland habitats in Europe thanks to traditional cattle and sheep grazing.

C. Kiskunság—located in central Hungary in the Danube–Tisza Interfluve (part of the Great Hungarian Plain), the second largest saline habitat complex in the Pannonian Basin ranks first in variability, as all types of saline/alkali habitats are represented in local variations [31]. Until river regulations began at the end of the 19th century, the Danube regularly flooded its 20–30-km-wide flood plain, which enabled the formation of large sodic lakes of high natural importance [32]. Typical soils are calcareous Solonchak and Solonchak-Solonetz, covered mainly by the annual vegetation of *Therosalicornietea* and *Crypsietea aculeatae*, while perennial halophytic stands of *Puccinellion limosae*, *Festucion pseudovinae*, and *Scorzonero-Juncion gerardii* are also well-developed. Today, the area is a national park with dominating rangelands managed by cattle and sheep grazing.

Each of these three regions has a moderately continental climate, with hot and dry summers and mild winters. The annual precipitation is 500–550 mm and the annual average temperature is 10.5 °C [33].

### 2.2. Study Design

#### 2.2.1. Selection of Study Objects

The target habitat Pannonic salt steppes is a mosaic of several grassland formations reflecting different soil salinity and water regime depending on the micro-elevation of the terrain [7,34]. Three typical halophytes (flagship species for conservation) were chosen to represent the target habitat:

*Artemisia santonicum* subsp. *patens* K. M. Perss (later on *A. santonicum*), *Asteraceae*, is a perennial species of the Eurasian continental steppes on sodic Solonetz/sodic soils. From an ecological viewpoint, its vegetation optimum is in the association *Artemiso-Festucetum pseudovinae* (alliance *Festucion pseudovinae*) where it is a diagnostic, constant and characteristic taxon [22,25,27]. *A. santonicum* is a Red List species, and on its northernmost distribution limit (Slovakia, Austria) is evaluated in the endangered category of the International Union for Conservation of Nature and Natural Resources (IUCN) [35,36]. This species indicates habitat *Artemisia* steppes within target habitat Pannonic salt steppes [37].

*Camphorosma annua* Pall. (later on *C. annua*), *Amaranthaceae*, is an annual species forming initial monodominant stands in shallow depressions of the natural Solonetz habitats. It occupies barren spots with the highest soil salinity within the salt steppes zonation [25,38]. In Slovakia, on its northernmost occurrence, it is critically endangered according to the IUCN Red List [36], and on its western distribution border (Austria and Croatia) it is endangered [35,39]. It is the indicator species of the annual salt pioneer swards (association *Camphorosmetum annuae*, alliance *Puccinellion limosae*) within the target habitat [37].

*Tripolium pannonicum* (Jacq.) Dobrocz. subsp. *pannonicum* (later on *T. pannonicum*), *Asteraceae*, is an annual, biennial, or shortly perennial species of shallow waterlogged depressions on saline and sodic soils. The main species *T. pannonicum* is very variable. Two subspecies were described: *T. pannonicum* subsp. *pannonicum* and *T. pannonicum* subsp. *tripolium*. In Europe, the nominate subspecies occurs in Spain, Germany, the Pannonian Basin, and south eastern Europe while subsp. *tripolium* is reported along most of the sea coasts from Europe and Asia [40]. It is diagnostic, characteristic, and constant species of the association *Puccinellietum limosae* [27]. It has the widest coeno-ecological preferences among the indicator species, occurring in Solonchak salt marshes of the order *Scorzonero-Juncion geradii*, in the littoral zone of sodic lakes of the class *Crypsieta aculeatae* [38] and in brackish marshes of the class *Phragmito-Magnocaricetea* [37]. In Slovakia it grows mostly in the vegetation of *Puccinellion limosae*. It is categorised as endangered in Slovakia [36] and Austria [35], while it is critically endangered in the Czech Republic [41]. This species indicates dense and tall *Puccinellia* swards [37] of the target habitat.

These obligate halophytes were selected as flagship species having high indication value of natural salty areas compared to other halophytes in the Pannonian Basin and are relatively common in the study area. *C. annua* and *A. santonicum* are considered as diagnostic species, together with *T. pannonicum* as constant species of Pannonic salt steppes [42]. None of them are spreading along roadsides benefiting from road salting as are *Puccinellia distans* agg. or *Spergularia salina* [43,44].

#### 2.2.2. Vegetation Sampling

The field survey was conducted between 2016 and 2018 in the second half of the growing season. To determine the ecological range limits of the indicator species of Pannonic salt steppes (where they are able to grow from optimal to suboptimal conditions in their target habitat), we conducted a plot-based vegetation survey. Data on fine scale vegetation composition, land use type (grazing, mowing, or abandonment—obtained from local experts), and soil properties were collected.

Plots with a uniform size of 0.5 × 0.5 m were sampled altogether on 35 different sites throughout the Pannonian Basin; on the norther distribution border of salt steppes in Slovakia (Podunajská nížina) and in their central distribution in Hungary (Hortobágy and Kiskunság). The plots were positioned within homogenous areas bearing the defined indicator species. On the Slovak sites, we recorded the plots in the whole range of habitat naturalness from preserved to degraded stages (for *A. santonicum* 41 plots, for *C. annua* 26 plots, and for *T. pannonicum* 34 plots); on the Hungarian sites, we sampled plots of pristine Pannonic salt steppes serving as reference (10 plots for *A. santonicum* and 13 plots for *C. annua* in Hortobágy, 10 plots for *T. pannonicum* in Kiskunság). Altogether 134 plots were recorded and analyzed.

The recording was conducted on two scales. The percentage cover of vascular plants was estimated on the whole plot area (0.5 × 0.5 m). For frequency data, the plot was divided into 25 subplots (size 0.1 × 0.1 m) using a wire (see photographs in Supplementary Material S2) where the presence/absence data of vascular plants were recorded. Plot selection was semi-random due to the characteristic patchiness of inland halophytic habitats. To avoid vegetation heterogeneity, we placed the sampling wire in homogenous stands of the target vegetation types of the studied species, reflecting the characteristic species composition according to the actual habitat classification of inland saline vegetation of the Pannonian Basin [37].

#### 2.2.3. Soil Sampling

At the time of vegetation recording, one soil sample was taken per plot at a depth of 0–12 cm. Altogether 77 samples were collected, 25 in the vegetation with *A. santonicum* (15 from Slovakia, 10 from Hortobágy), 21 in *C. annua* (11 from Slovakia, 10 from Hortobágy), and 31 in *T. pannonicum* (21 from Slovakia, 10 from Kiskunság).

They were subjected to laboratory analysis of the following properties: EC (electric conductivity), SAR (sodium adsorption ratio), Na_exch (exchangeable sodium), pH, Cox (organic carbon), and Ntot (total nitrogen). The pH and EC were determined by the method of Sotáková et al. [45]. A saturated soil extract was obtained by centrifugation, and pH and conductivity were determined in the supernatant using a pH electrode and conductometer, respectively. Exchangeable cations (Na_exch) were determined according to ISO 13536. The exchangeable cations were displaced by barium chloride buffered at pH 8.1. The concentration of displaced cations was determined by F-AAS. For calculating SAR, concentrations of Ca^2+^, Mg^2+^, and Na^+^ (determined by F-AAS in the saturated soil extract) were used. Simultaneous determination of carbon and nitrogen (Cox and Ntot) was carried out by dry combustion. First, soil samples were dispensed into the combustion tube. The result of combustion is a mixture of gases, nitrogen oxides, CO_2_, H_2_O, SO_2_, and the excess oxygen. The mixture of gases was passed through the reduction catalyst, which eliminates redundant oxygen and reduces the nitrogen oxides to nitrogen alone. This mixture was split on a thin-layer chromatography column and analyzed by a thermal conductivity detector. This method also determines levels of inorganic carbon originating from CaCO_3_, NaHCO_3_, etc. In order to exclusively measure Cox concentration, inorganic carbon must be removed by decalcification using a dilute HCl solution. The analytical database (Supplementary Material S3) was checked for consistency according to the suggestions of Richards [46].

#### 2.2.4. Data Processing

The plot dataset of 134 relevés (cover scale) and the subplot dataset of 3350 relevés (presence/absence) were stored in the TURBOVEG database [47] and exported for analysis in the JUICE program [48]. The recorded species were divided into characteristic species (including indicator species), accompanying halophytic species, and generalists according to the list of halophytes compiled by V. Krist [49] from the northern part of the Pannonian Basin. Details of classification criteria are contained in Tables S1–S3 in Supplementary Material S1. Characteristic species have a percentage frequency in reference plots of at least 5%. For this purpose, the scale of 0.01 m^2^ was used. Due to the higher number of records, this scale captures the typical species composition more exactly and suppresses random occurrences of species with low cover (frequency below 5%), which would otherwise be recorded at the 0.25 m^2^ scale (for details, see Figures S1–S3 in Supplementary Material S1).

The recorded plots were divided into five groups of naturalness, based on the species composition of the three surveyed plant communities of Pannonic salt steppes (*Artemiso-Festucetum pseudovinae, Camphorosmetum annuae*, and *Puccinellietum limosae*) represented by the three indicator species. Classification criteria are shown in Table 1.

Group 1: vegetation of the highest naturalness, typical association of the indicator species, high representation of specialists, no occurrence of species indicating degradation (generalists).

Group 2: vegetation of high naturalness, typical association of the indicator species, only random occurrence of generalists.

Group 3: vegetation with decreased naturalness and minor signs of degradation, not typical association of the indicator species, low proportion of generalists.

Group 4: degraded vegetation of the association of the indicator species with high representation of generalists.

Group 5: strongly altered vegetation not assignable to any target association, generalists predominate.

#### 2.2.5. Data Analysis

**Figure 2:**
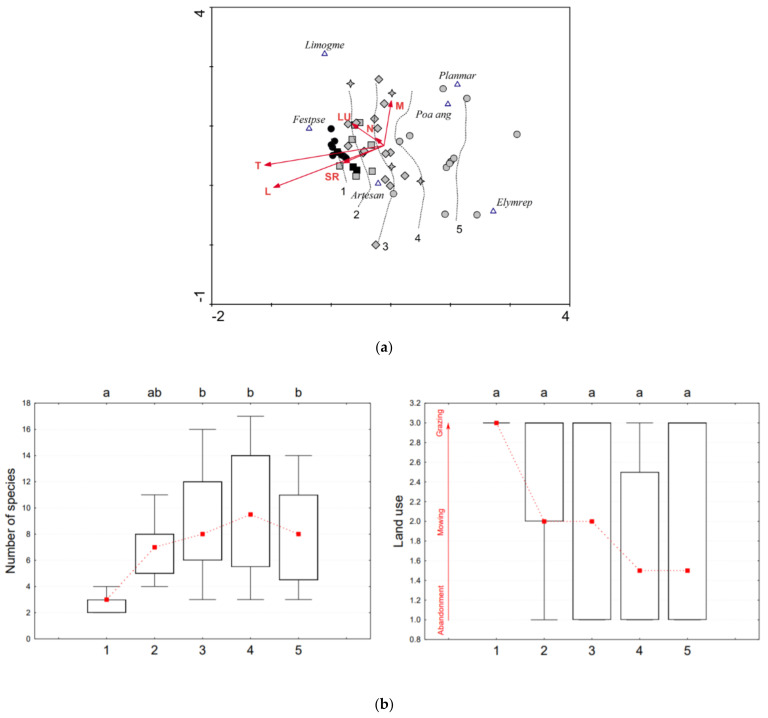
(**a**) Data attribute plot of vegetation naturalness for indicator species *Artemisia santonicum* subsp. *patens* based on the LOESS visualization method and displaying Borhidi indicator values [51] as vectors. Detrended correspondence analyses (DCA) ordination diagram projects 51 records/plots classified according to vegetation naturalness groups 1 to 5: (black circle ●—group 1 in Hungary, black square ■—group 1 in Slovakia, grey square ■—group 2, grey diamond ♦—group 3, grey star ✦—group 4, grey circle ●—group 5) and six species: *Artesan*—*Artemisia santonicum, Elymrep*—*Elytrigia repens, Festpse*—*Festuca pseudovina, Limogme*—*Limonium gmelinii, Planmar*—*Plantago maritima*, and *Poaang*—*Poa angustifolia*. Displayed vectors: T—temperature; L—light; SR—soil reaction; N—nutrients; M—moisture; LU—land use. Ordination scores of the displayed species (species weight range) are more than 0.10. Axis eigenvalues: 0.534 and 0.349; DCA 1 axis length: 3.091; DCA2 axis length: 2.963. (**b**). Pair-plot (boxplots of species number and land use intensity) in five groups of naturalness (1–5) with connected median points for *Artemisia santonicum* subsp. *patens*. The upper letters (a, b) indicate homogeneous groups according to Tukey’s post-hoc test (α = 0.05).

**Figure 3:**
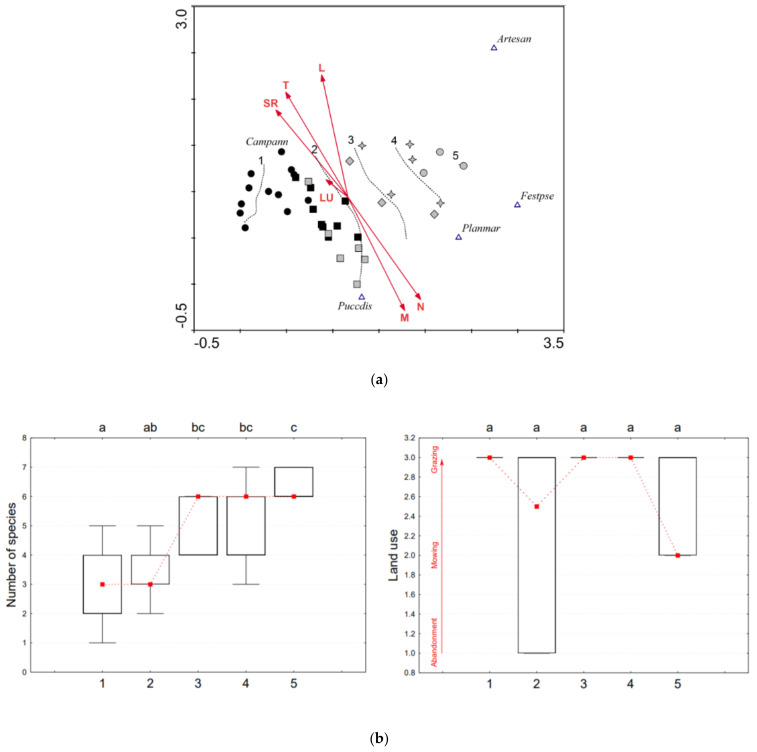
(**a**) Data attribute plot of vegetation naturalness for indicator species *Camphorosma annua* based on the LOESS visualization method and displaying Borhidi indicator values as vectors. DCA ordination diagram projects 39 records classified according to vegetation naturalness (black circle ●—group 1 in Hungary, black square ■—group 1 in Slovakia, grey square ■—group 2, grey diamond ♦—group 3, grey star ✦—group 4, grey circle ●—group 5) and six species: *Artesan*—*Artemisia santonicum, Campann*—*Camphorosma annua, Festpse*—*Festuca pseudovina, Planmar*—*Plantago maritima and Puccdis*—*Puccinellia distans* agg. Displayed vectors: T—temperature; L—light; SR—soil reaction; N—nutrients; M—moisture; LU—land use. Ordination scores of the displayed species (species weight range) are more than 0.10. Axis eigenvalues: 0.467 and 0.136; DCA 1 axis length: 2.413; DCA2 axis length: 1.511. (**b**). Pair-plot (boxplots of species number and land use intensity) in five groups of naturalness (1–5) with connected median points for indicator species *Camphorosma annua*. The upper letters (a, b, c) indicate homogeneous groups according to Tukey’s post-hoc test (α = 0.05).

**Figure 4:**
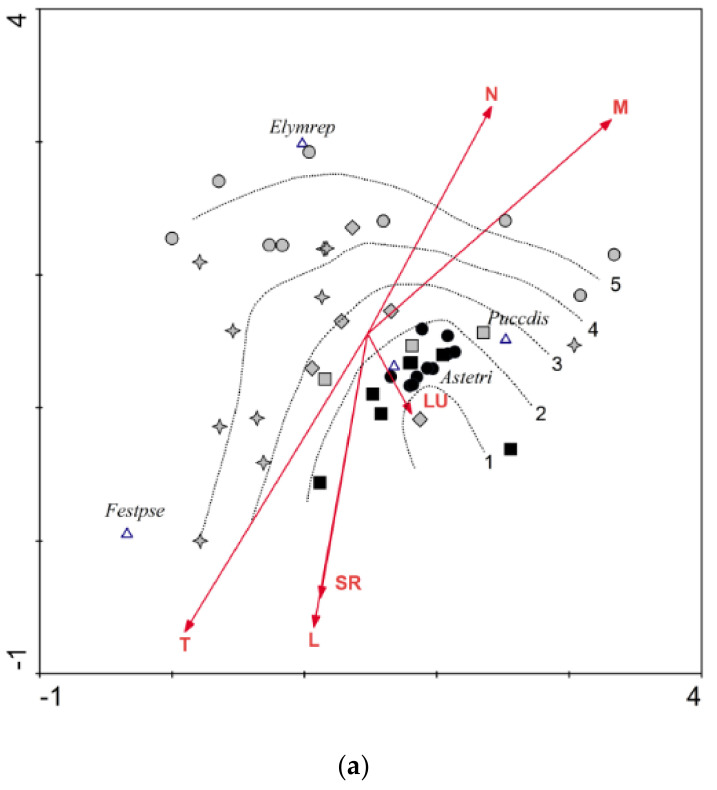

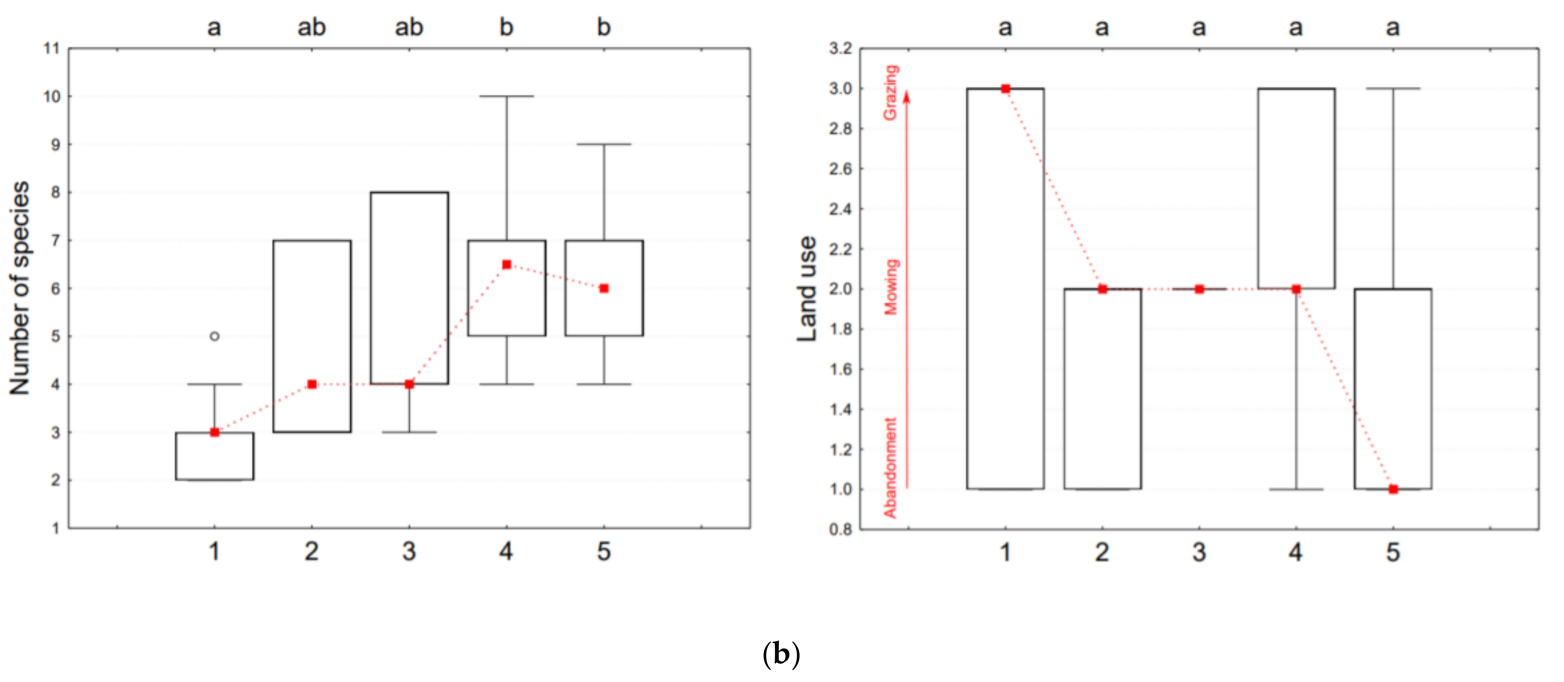
(**a**) Data attribute plot of vegetation naturalness for indicator species *Tripolium pannonicum* subsp. *pannonicum* based on the LOESS visualization method and displaying Borhidi indicator values as vectors. DCA ordination diagram projects 44 records classified according to vegetation naturalness (black circle ●—group 1 in Hungary, black square ■—group 1 in Slovakia, grey square ■—group 2, grey diamond ♦—group 3, grey star ✦—group 4, grey circle ●—group 5) and six species: *Astetri*—*Tripolium pannonicum, Elymrep*—*Elytrigia repens, Festpse*—*Festuca pseudovina,* and *Puccdis*—*Puccinellia distans* agg. Displayed vectors: T—temperature; L—light; SR—soil reaction; N—nutrients; M—moisture; LU—land use. Ordination scores of the displayed species (species weight range) are more than 0.10. Axis eigenvalues: 0.551 and 0.415; DCA 1 axis length: 3.262; DCA2 axis length: 2.789. (**b**). Pair-plot (boxplots of species number and land use intensity) in five groups of naturalness (1–5) with connected median points for *Tripolium pannonicum* subsp. *pannonicum*. The upper letters (a, b) indicate homogeneous groups according to Tukey’s post-hoc test (α = 0.05).

For each plot with a percentage cover scale, indicator values for light, temperature, moisture, soil reaction, and nutrients were calculated and weighted by species cover. Taking into account the strong representation of Pannonian species, indicator values appropriate for East-Central Europe were used by Borhidi [50]. Ecological indication values express optimum ecological niche of plant species on the gradient of nutrients, moisture, soil reaction, continentality, temperature, light, and salinity on the ordinal scale (mostly 1–9, possibly 1–12 for humidity). They are often used to rapidly estimate site conditions from species composition when measured values of environmental variables are not available. Values are based on field observations, and partly on evidence from ecological experiments and measurements of environmental variables [51]. Ecological indication values of species recorded on our plots are contained in Supplementary Material S3. They were used in the detrended correspondence analyses (DCA, Figure 2a, Figure 3a, and Figure 4a).

The species number and land use type in the groups of naturalness and analyzed soil properties were displayed by boxplots, for species number and land use with connected median points; all were generated in the program Statistica 7 [52]. The type of land use (surveyed at the time of vegetation sampling) was scaled according to its strength of positive effect on the vegetation based on general studies about management of semi-natural grasslands [17,53]: 3—extensive grazing by grey cattle/sheep/mixed herd (land use type at moderate intensity, positive effect), 2—one-year mowing (low intensity, mixed effect), 1—abandonment (zero intensity, negative effect). Statistically significant differences among groups displayed in boxplots were tested using ANOVA and for species number and land use also Tukey post-hoc test (α = 0.05).

For the ordination (DCA and data attribute plots with the LOESS visualization regression modelling method), we used the program CANOCO for Windows 4.5 [54]. The map was created using the program QGIS, version 3.2 [55] with the QuickMapServices plugin and a terrain background layer from Stamen Design with data by OpenStreetMap.

## 3. Results and Discussion

### 3.1. Ecological Range Limits of Indicator Species of Pannonic Salt Steppes—Vegetation Composition and Land Use Approach

#### 3.1.1. *Artemisia santonicum* Subsp. Patens (ART)

Three plots (from the 41) in Slovakia had the highest naturalness and they overlapped with the 10 reference plots from Hortobágy, Hungary (Figure 2a). Those records are plotted on the left side of the DCA diagram, with the highest preference for the indicator values temperature, light, soil reaction, and land use (Figure 2a, Supplementary Material S3) which refers to more extreme habitat conditions, optimal for saline vegetation. The majority of the Slovak plots have higher moisture and nutrient values (Figure 2a) which refers to decreased habitat extremity, suboptimal for salt steppes.

In group 1 the average species number was three; in the rest of plots there were eight (Figure 2b, left). The highest number of species was found in groups 2, 3, and 4; in the most degraded stands (group 5) the expansion of generalists hindered the occurrence of other species. The difference in absolute species number in the case of ART was outstanding: 68 species were recorded on the Slovak plots and 7 in Hortobágy (Table S1 in Supplementary Material S1). In stands of reduced naturalness (groups 2–5), the most frequent generalists were *Elytrigia repens* (30%), *Poa angustifolia* (26%), then *Achillea millefolium*, *Inula britannica*, and *Bromus hordeaceus*.

Regarding land use (Figure 2b, right), the vegetation degradation was related to poor management. Most of the group 5 plots were recorded on abandoned sites, and only a few of them were grazed (Supplementary Material S3).

A substantial aspect in *Artemisia* steppes is the proportion of the two main association species (*A. santonicum* and *Festuca pseudovina*). Preserved sodic steppes are characterized by high cover of F. pseudovina, generally 40–70% [56]. While the percentage cover of *A. santonicum* in both areas was similar (65% versus 64%, see Table S1 in Supplementary Material S1), *F. pseudovina* showed great differences: in Hungary 97% and in Slovakia 55%. This suggests that *F. pseudovina* is suppressed by expansive grasses in degraded salt steppes. *A. santonicum* persists in unfavorable conditions longer than *F. pseudovina* and the former vegetation of *Artemisia* steppes is indicated only by the remnant populations of *A. santonicum*, and the characteristic subordinated species are missing. Plots with lower naturalness revealed that the survival chance of this species is high. During our field survey one-meter high specimens of *A. santonicum* have been observed occupying a 40-year-old poplar plantation without any characteristic salt steppe species (site no. 11 in Supplementary Material S3). *A. santonicum* turns out to be a stress tolerant competitor (C-S strategy) [57]: on strongly salt-affected soils it uses mechanisms of resistance to high salt content, while on weaker saline soils its perennial life form (hemicryptophyte) as well as absolute height of individuals is successfully used in competition with glycophytes. It tends to form pure stands on soils with high salinity after destruction of the vegetation cover (e.g., former ploughing). Due to the deep rooting its great regeneration ability was confirmed also by Galvánek et al. [58]. After all, the recent populations’ vitality did not correspond with the habitat quality. We recorded the most vital populations of the species in groups 2, 3, and 4. These are previously plowed (about 20 years ago) and subsequently abandoned salt steppes. High abundance of the indicator species cannot be associated with good habitat condition.

#### 3.1.2. Camphorosma Annua (CAM)

Nine plots (from the 26) of Slovakia had the highest naturalness similar to the 13 reference ones sampled in Hortobágy (Supplementary Material S3), which are open, extremely dry, nutrient-poor soils with high soil reaction and active land use (grazing). Those plots are located on the left side of the DCA (Figure 3a). In the lower part of the ordination space are plots with higher moisture; these are regarded as intact vegetation of *Puccinellietum limosae*. In the right section are plots of drier habitats (transition to *Artemiso-Festucetum pseudovinae*) which suggest that plots of naturalness groups 4 and 5 cannot be regarded as association of *Camphorosmetum annuae*.

In reference plots, *C. annua* had altogether four accompanying species, mostly *Plantago tenuiflora* and *Matricaria chamomilla* (Table S2 in Supplementary Material S1). They are typical association species with similar ecological preferences as *C. annua* [27], but in plots of group 1 in Slovakia were not recorded at all. On the DCA (Figure 3a) these plots do not overlap with the Hungarian reference ones. The perennial halophytes in groups 2–5 (*Puccinellia distans* agg. 63%, *A. santonicum* 21%, and *F. pseudovina* 21%, see Table S2 in Supplementary Material S1) indicate the succession of the pioneer association towards closed stands of *Artemisia* steppes as a result of decreasing habitat extremity. It is also associated with the presence of generalists; 10 different species were recorded, most frequently *Poa angustifolia* and *Cynodon dactylon*. The absolute species number was 17. The lowest mean species number (three) was detected in stands of group 1; the highest (six) was detected in group 5 (Figure 3b, left), which gives a different pattern as in the case of ART (Figure 2b, left). Here species number is increasing with decreasing naturalness and the most degraded plots are not colonized by a certain generalist species.

Habitat quality is associated with the intensity of land use, and plots with the highest naturalness were all grazed (Figure 3b, right); however, in the case of CAM, specific ecological conditions were more crucial. As the influence of land use was not significant (*p* > 0.05), abiotic stress (high soil salinity and an unstable water regime) acted here as a stabilizing mechanism, maintaining the survival of this species. Stands with *C. annua* have managed to persist through long-term abandonment in Slovakia due to extreme edaphic conditions. Sites where *C. annua* stands occur refer to an overall higher habitat quality. The absence suggests worsened habitat quality of Pannonic salt steppes, since a certain vegetation zone of salt steppes indicates the most extreme habitats are missing.

Long term survival chances of *C. annua* are therefore limited compared to *A. santonicum* due to the higher preference for habitat extremity and very low competition ability. A higher salt demand by halophytes results in lower competitiveness in non-saline environments as it was formerly described by Ungar [59] and observed also by Grigore et al. [60]. Species with high bioindicator potential are only able to survive in habitats with narrowly defined physicochemical characteristics [61].

#### 3.1.3. *Tripolium pannonicum* Subsp. *pannonicum* (TRI)

Seven plots (from the 34) in Slovakia were classified as having the highest naturalness and there was a partial overlap with the 10 reference plots recorded in Kiskunság (Hungary). Land use vector on Figure 4a points in the direction of the group-1 plots. On the Hungarian plots, six species were recorded (Table S3 in Supplementary Material S1) each typical for the target association *Puccinellietum limosae*. High frequency was observed for *T. pannonicum* and *P. distans* agg. (99.6% and 89.6%). On the Slovak plots, *T. pannonicum* had only 68% frequency and *P. distans* 35%; moreover, 47 species were found altogether, with a strong prevalence of generalists (Table S3 in Supplementary Material S1). Mean species number increased significantly in vegetation of lower naturalness (Figure 4b, left). Similar to that observed for ART, the maximum species number was found in moderately degraded vegetation; on the most degraded plots it decreased due to expansion of generalists like *E. repens* (26%), *P. angustifolia* (15%), and, to a lesser extent, *Agrostis stolonifera* and *I. britannica* (Table S3 in Supplementary Material S1).

The relation between naturalness and land use is the same for TRI as for ART and CAM: reduced management intensity resulted in greater degradation of the vegetation of the studied species (Figure 4b, right). In Slovakia, the vegetation most similar to the reference plots (high cover of characteristic species and low species number) is of secondary origin, regenerated after shallow tillage of flat waterlogged depressions. Although abandoned, the soil disturbance has inhibited secondary succession. In the case of TRI, the most vital populations were also found on ploughed plots, similar to ART. The species has good competition ability on disturbed saline soils although its dominance is only temporary as was also observed by Melečková et al. [62]. The characteristic subordinated species typical in reference area Kiskunság were missing there.

In spite of the statistically insignificant difference between the five groups of naturalness from the aspect of land use, Figure 2b (right), Figure 3b (right), and Figure 4b (right) show a clear trend of decreasing intensity of management at higher degradation level. A more pronounced significance of this trend is limited by the low number of existing sites in Slovakia. Almost all of the recorded plots in Hungary were maintained by grazing; in Slovakia, management measures were heterogeneous. Of the overall 101 plots, 39 were grazed, 28 were mowed, and 34 were abandoned (Supplementary Material S3). Grazing has been re-introduced at several sites only in the last decade, which has facilitated the living conditions of indicator species, but their indigenous plant communities have not been restored.

### 3.2. Soil Chemistry of Indicator Species of Pannonic Salt Steppes (ART, CAM, TRI)

**Figure 5:**
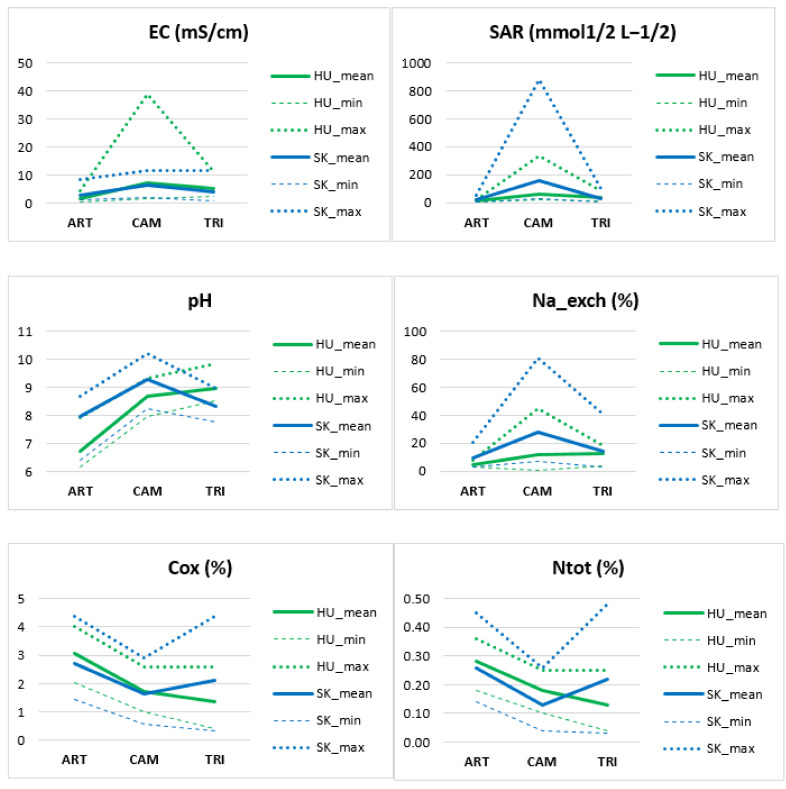
Minimum, maximum, and mean values of soil chemical properties measured on 77 vegetation plots.

Soil samples of vegetation with *Camphorosma annua* had the most extreme edaphic conditions: the highest measured EC, SAR, Na_exch, and pH which indicate strongly sodic soils (Figure 5). Out of the total 77 soil samples, 40 had EC higher than 4 mS/cm and pH higher than 8.5, which are classified as sodic and saline soils according to [63]. From there, 18 samples were detected in the *C. annua* plots (groups of naturalness 1 to 4), 18 samples were detected in the plots of *Tripolium pannonicum* in each group of naturalness, and four samples were detected in the plots of *Artemisia santonicum* (groups of naturalness 1 to 3) (Supplementary Material S3).

**Figure 6:**
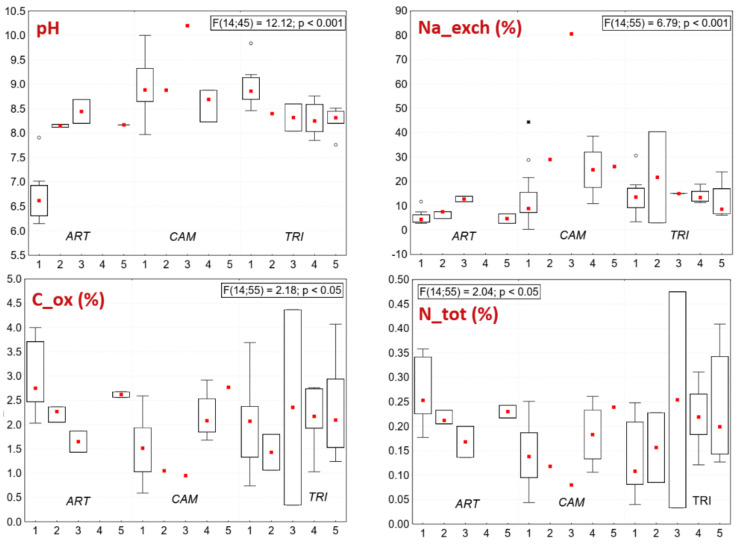
Significant soil properties for three indicator species (ART, CAM, and TRI) at different groups of naturalness (1–5): Na_exch—exchangeable sodium; pH—soil reaction; Cox—organic carbon; Ntot—total Nitrogen. Boxplots show median (red squares), interquartile range (boxes), non-outlier range (whiskers), outliers (empty circles), and extreme values (asterisk). Statistical significance by ANOVA.

It follows that the lowest sodicity had plots of ART (Figure 5 and Figure 6). It refers to less extreme edaphic conditions in the *Artemisia* steppe zone which is in accordance with the general knowledge about the ecology and zonation of Pannonic salt habitats [7]. The highest nutrient content (Ntot and Cox) was detected in ART and TRI, the lowest in CAM (Figure 5 and Figure 6), which is related to limited nutrient availability in saline soils [64]. Soils of TRI plots had a high variance of Ntot and Cox, and the species was able to grow on nutrient-poor and nitrogen-rich soils as well.

On several sites with degraded vegetation we detected higher sodicity (higher Na_exch, SAR, and pH, see Figure 5) than in the reference salt steppes of Hungary. We explain this unexpected finding by the impact of past ploughing. In pristine Solonetz steppes, the topsoil layer, where salinity and pH are usually lower than at deeper levels, is leached [65], as Figure 6 similarly demonstrates in plots of ART, group 1, pH. When such a soil type is rotated by ploughing, the deeper layer is exposed to the surface. As it is more clayey and sodic than the topsoil, the salts will leach more slowly and the new secondary sodic soil surface is again occupied by halophytes. Simultaneously, the disturbed surface favors the colonization of opportunistic weeds [58] which in case of abandonment remain a constant component of the regenerated vegetation. The plots in Slovakia demonstrate this phenomenon. Every site of Pannonic salt steppes has there undergone an alteration of the soil due to attempts to fertilize the salt-affected areas. They have been ploughed at least once, which caused an overturn of the soil profile and changes in soil properties. For this reason, the typical plant-soil correlations detected, for example by Tóth and Rajkai [66], have not been confirmed recently in Slovakia. Our samples taken from the topsoil (at a depth of 12 cm) are not deep enough to demonstrate the effect of soil rotation. Soil analyses were therefore not straightforward in evaluating the current condition of the target habitat, compared to the vegetation or land use approach. However, they revealed the whole range of key soil properties in which the studied indicator species and their target vegetation persist from favorable (optimal) to marginal (suboptimal) ecological conditions.

### 3.3. Current Condition of Indicator Species of Pannonic Salt Steppes

A small proportion of plots (19%) in Slovakia overlapped with the Hungarian reference ones, having the highest naturalness (group 1). The majority (57%) of plots had decreased naturalness and 24% of plots were detected in the lowest naturalness (group 5). Their small area within each site points to reduced habitat quality compared to Hungarian sites where each vegetation type was found in larger areas in a relatively high habitat quality [19]. In Slovakia, salt steppes of high naturalness are limited only to a few sites and the long-term survival chance is limited due to fragmentation and accelerated vegetation degradation.

The most threatened ones are barren spots of *Camphorosmetum annuae* and its indicator species, *C. annua*. In its eight known localities at its absolute northern distribution limit, there is no population where its area exceeds 50 m^2^. On the other hand, the disappearance of the community in its central area is local, for example in Kisalföld (northwestern Hungary) close to the study area Podunajská nížina where an overall decreased habitat quality has been reported [19,67]. Being a border floristic element is therefore a hindering factor for species survival.

Regarding *A. santonicum* and *T. pannonicum*, their vital populations in Slovakia are mono-dominant stands lacking specialists typical for their associations. They are able to co-exist with generalists (mainly *A. santonicum*, described also by Bölöni et al. [37]), but their seemingly good condition is temporary. Abundance of these indicator species therefore cannot be linked with habitat naturalness.

### 3.4. Conservation Perspectives of Pannonic Salt Steppes on Their Border Area

The ecological circumstances (vegetation composition and land use) of *A. santonicum*, *C. annua*, and *T. pannonicum* reflect well the current habitat quality of Pannonic salt steppes. Worsened environmental conditions (enrichment of generalist species in the vegetation and lack of habitat management) pointed to decreased habitat quality at the northern distribution limit of the target habitat.

Natural habitats of halophytes deteriorate faster in abandoned vegetation due to the high pressure of spreading generalists than on sites where soil was formerly ploughed [62]. Therefore, the introduction of grazing is the most desired restoration measure for their conservation as phytomass consumption and seed dispersal promote the emergence/survival of specialists. A positive example is the rediscovery of *Triglochin maritima* in salt marshes of Slovakia, a rare species in the Pannonian Basin which was found on trampled salt depressions after 60 years [68]. An intended consequence of grazing was to improve the survival conditions of *A. santonicum*, *C. annua*, and *T. pannonicum* in Slovakia. Those stands are, however, not in the optimal condition seen in the center of their distribution. Populations of these species are well maintained, but restoration of their characteristic plant communities is unfeasible even at the most appropriate grazing intensity. The prospect of reversing the degradation processes is more promising in contiguous areas than in the highly fragmented salt habitats on their distribution border.

## 4. Conclusions and Practical Outcomes

The habitat quality of Pannonian salt steppes at their northern geographical limit is decreased compared to their central occurrence. Only 19 out of the 101 plots were detected at the highest naturalness. The recent sites are scattered islands inside the intensively used landscape and halophytic specialists are substituted by generalists (*Elytria repens*, *Agrostis stolonifera*). Suboptimal ecological conditions which increase their risk of extinction may be mitigated by appropriate management in the case of *Artemisia santonicum* and *Tripolium pannonicum* subsp. *pannonicum*, where wider niche and competition ability was detected. The occurrence of the therophytic, low competitor *Camphorosma annua* was driven largely by abiotic stress; grazing alone is insufficient for its long-term persistence in decreased habitat extremity.

With respect to the groups of naturalness, soil analyses brought disparate results. Contrary to expectations, higher habitat naturalness did not correspond with habitat extremity (high soil sodicity). Each indicator species in its target vegetation was detected on less sodic soils, too, regardless of the level of naturalness. We assume that *C. annua, A. santonicum*, and *T. pannonicum* are able to grow on soils of greatly decreased salinity/sodicity if competitor species (generalists) are absent. Even on the reference vegetation in Hungary were detected low rates of SAR, Na_exch, EC, and pH (see Figure 5 for minimal values). This refers also to the high stability of salt steppes there thanks to the continuous non-intensive grazing. On Slovak sites the abiotic stress takes the major role in the persisting of abandoned or short-time grazed salt steppes.

The plot-based vegetation analyses (beyond presenting the optimal and marginal synecological range limits of indicator species) can serve as a quick tool for assessing the overall condition of the priority habitat 1530 Pannonic inland salt steppes. We proceeded from the definition of the Habitats Directive [69] which states that a site is of good quality if it allows a good chance of survival for a range of species that are typical for the habitat. The presented method may be used as a basic material in conservation practice, possibly to apply to different non-forest habitats in any other geographic area. Accurate habitat assessment based on remote sensing on a micro-regional scale [70] and the increasing efficiency of drones [71] has high accuracy, but their time and technological demand show some limitations which are difficult to overcome when the main goal is to obtain complete data in a short time. Evaluating habitat conservation status still relies on traditional fieldwork.

## Figures and Tables

**Table 1:** Classification criteria into five groups of naturalness based on the proportion of characteristic, accompanying halophytic and generalist species (for supporting data see S1).

% Cover of the 0.05 × 0.05 m Plot	Group No. and Its Symbol in Figure 2a, Figure 3a and Figure 4a				
	1 ● ■	2 ■	3 ♦	4 ✦	5 ●
Characteristic species, including indicator species	≥50%	≥50% (min. two species)	≥50%	(<50% *) ≥50%	
Generalists indicating degradation	0%	>0% to <5%	>0% to <25%	(>0%*) ≥25% to <50%	≥50%
Accompanying halophytic species				≥30% *	

* If the characteristic species do not reach 50% cover, but the accompanying halophytes have cover higher than 30% (e.g., atypical occurrence of *Tripolium pannonicum* in degraded plots of the association *Artemisio-Festucetum pseudovinae*).

## Data Availability

All data analyzed during this study are contained within the article’s supplementary material [S1, S2, S3]

## Supplementary Materials

The following are available online at https://www.mdpi.com/2223-7747/10/3/530/s1, S1_Supporting data for calculating habitat naturalness (groups 1–5) described in Methods (it contains Tables S1–S3 and Figures S1–S3). S2_Photographs of vegetation plots with three flagship species of salt steppes taken by the authors during field sampling. S3_Complete list of recorded plots with attributes.
