# Supplementary material for: Current Condition of Pannonic Salt Steppes at Their Distribution Limit: What Do Indicator Species Reveal about Habitat Quality?
Source: Plants (Basel). 2021 Mar 11;10(3):530. doi: 10.3390/plants10030530 (PMC7999981; doi:10.3390/plants10030530)

Electronic supplementary material of manuscript

Title:

**Current condition of Pannonic salt steppes at their distribution limit: what do indicator species reveal about habitat quality?**

by Zuzana Dítě, Róbert Šuvada, Tibor Tóth, Pavol Eliáš, Vladimír Píš & Daniel Dítě

submitted to PLANTS journal

**Supplementary material S1.** Supporting data and additional analyses for exact determination of habitat quality (groups 1, 2, 3, 4 and 5) based on the proportion of characteristic, accompanying and generalist species of the three surveyed indicator species of Pannonic salt steppes.

**Table S1.** Percentage frequency of all recorded vascular plant species in vegetation of *Artemisio-Festucetum pseudovinae* (indicated by *Artemisia santonicum* subsp. *patens*) in different scales (0.01 m^2^ and 0.25 m^2^) surveyed in Slovakia (Podunajská nížina) and on reference plots in Hungary (Hortobágy). All species were classified to characteristic species, including indicator species (first line, **bold**), accompanying halophytic species (second line, *italics*) and generalists indicating degradation (third line).

| *Artemisia santonicum* plots | **Hungary** | | **Slovakia** | |
| --- | --- | --- | --- | --- |
|  | 250 subplots | 10 plots | 1025 plots | 41 plots |
|  | 0.01 m^2^  Percentage frequency | 0.25 m^2^  Percentage frequency | 0.01 m^2^  Percentage frequency | 0.25 m^2^  Percentage frequency |
| **Festuca pseudovina** | **96.8** | 100.0 | 54.8 | 70.7 |
| **Artemisia santonicum** | **64.4** | 100.0 | 65.0 | 100.0 |
| **Scorzonera cana** | **5.2** | 40.0 | 8.0 | 41.5 |
| *Limonium gmelinii* | 3.6 | 20.0 | 6.9 | 22.0 |
| *Camphorosma annua* | 3.6 | 10.0 | 0.3 | 4.9 |
| *Atriplex littoralis* | 0.4 | 10.0 | 1.0 | 2.4 |
| *Plantago schwarzenbergiana* | 0.4 | 10.0 | 0.0 | 0.0 |
| *Plantago maritima* | 0.0 | 0.0 | 14.0 | 36.6 |
| *Puccinellia distans agg.* | 0.0 | 0.0 | 8.8 | 26.8 |
| *Tripolium pannonicum* | 0.0 | 0.0 | 5.5 | 24.4 |
| *Bupleurum tenuissimum* | 0.0 | 0.0 | 3.0 | 14.6 |
| *Hordeum geniculatum* | 0.0 | 0.0 | 3.2 | 7.3 |
| *Trifolium angulatum* | 0.0 | 0.0 | 3.4 | 7.3 |
| *Aster punctatus* | 0.0 | 0.0 | 1.0 | 4.9 |
| *Cerastium dubium* | 0.0 | 0.0 | 2.0 | 4.9 |
| Elymus repens | 0.0 | 0.0 | 30.9 | 48.8 |
| Poa angustifolia | 0.0 | 0.0 | 26.5 | 58.5 |
| Bromus hordeaceus | 0.0 | 0.0 | 13.1 | 43.9 |
| Inula britannica | 0.0 | 0.0 | 9.1 | 22.0 |
| Achillea millefolium | 0.0 | 0.0 | 7.9 | 26.8 |
| Cynodon dactylon | 0.0 | 0.0 | 3.9 | 12.2 |
| Lotus tenuis | 0.0 | 0.0 | 3.6 | 9.8 |
| Cerastium pumilum agg. | 0.0 | 0.0 | 3.4 | 17.1 |

* Other generalists, their percentage frequency on the scale of 0.01 m^2^ was lower than 3% in Slovakia, sorted by frequency value: Trifolium campestre, Vicia tetrasperma, Galium verum, Fragaria viridis, Atriplex prostrata, Agrostis stolonifera, Plantago major, Polygonum aviculare, Alopecurus pratensis, Bromus japonicus, Atriplex patula, Carex stenophylla, Arrhenatherum elatius, Tripleurospermum inodorum, Odontites vulgaris, Gypsophila muralis, Lepidium ruderale, Myosotis ramosissima, Plantago lanceolata, Carex hirta, Matricaria chamomilla, Veronica arvensis, Carex praecox, Dactylis glomerata, Lolium perenne, Myosurus minimus, Trifolium striatum, Daucus carota, Juncus compressus, Agrimonia eupatoria, Arenaria serpyllifolia, Carduus acanthoides, Cirsium arvense, Festuca arundinacea, Potentilla reptans, Taraxacum sect. Ruderalia, Trifolium repens, Allium vineale, Carex tomentosa, Centaurea jacea ssp. angustifolia, Conyza canadensis, Epilobium tetragonum, Lactuca saligna, Myosotis stricta, Vicia hirsuta.

**Fig. S1.** Horizontal bar plots of percentage frequency of species recorded in two different scales (0,01 m^2^ – red, 0,25 m^2^ – grey) on reference plots of Hungary in *Artemisia* steppes*.* On Axis X the scale break is marked at 5%, which is the limit for defining the characteristic species of particular vegetation type.


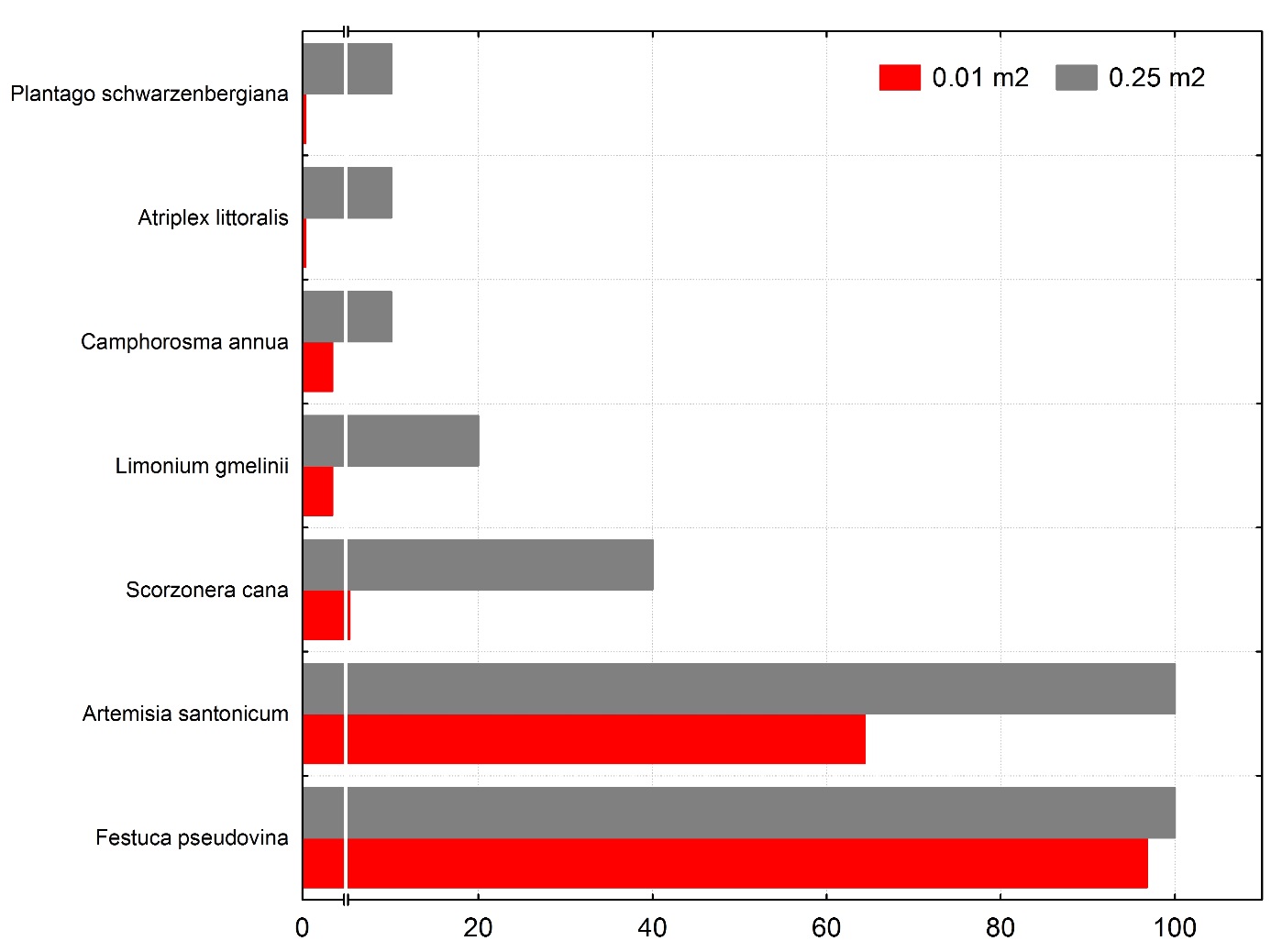


**Table S2.** Percentage frequency of all recorded vascular species in vegetation of *Camphorosmetum annuae* (indicated by *Camphorosma annua*) in different scales (0.01 m^2^ and 0.25 m^2^) surveyed in Slovakia (Podunajská nížina) and on reference plots in Hungary (Hortobágy). All species were classified to characteristic species, including indicator species (first line, **bold**), accompanying halophytic species (second line, *italics*) and generalists indicating degradation (third line).

| *Camphorosma annua* plots | **Hungary** | | **Slovakia** | |
| --- | --- | --- | --- | --- |
|  | 325 subplots | 13 plots | 1150 subplots | 26 plots |
|  | 0.01 m^2^  Percentage frequency | 0.25 m^2^  Percentage frequency | 0.01 m^2^  Percentage frequency | 0.25 m^2^  Percentage frequency |
| **Camphorosma annua** | **98.8** | 100.0 | 71.0 | 100.0 |
| **Puccinellia distans agg.** | **39.4** | 76.9 | 63.2 | 88.5 |
| **Matricaria chamomilla** | **36.0** | 53.8 | 0.0 | 0.0 |
| **Plantago tenuiflora** | **28.0** | 61.5 | 0.0 | 0.0 |
| *Cerastium semidecandrum* | 0.3 | 7.7 | 0.0 | 0.0 |
| *Tripolium pannonicum* | 0.0 | 0.0 | 0.3 | 3.8 |
| *Limonium gmelinii* | 0.0 | 0.0 | 0.4 | 11.5 |
| *Plantago maritima* | 0.0 | 0.0 | 9.6 | 61.5 |
| Artemisia santonicum | 0.0 | 0.0 | 21.4 | 42.3 |
| Festuca pseudovina | 0.0 | 0.0 | 21.4 | 30.8 |
| Bromus hordeaceus | 0.0 | 0.0 | 3.0 | 23.1 |

* Other generalists, their percentage frequency on the scale of 0.01 m^2^ was lower than 3% in Slovakia, sorted by frequency value: Cynodon dactylon, Poa angustifolia, Scorzonera cana, Trifolium campestre , Cerastium dubium, Elymus repens, Vicia sativa.

**Fig. S2.** Horizontal bar plots of percentage frequency of species recorded in two different scales (0,01 m^2^ – red, 0,25 m^2^ – grey) on reference plots of Hungary in vegetation of *Camphorosmetum annuae.* On Axis X the scale break is marked at 5%, which is the limit for defining the characteristic species of particular vegetation type.

**
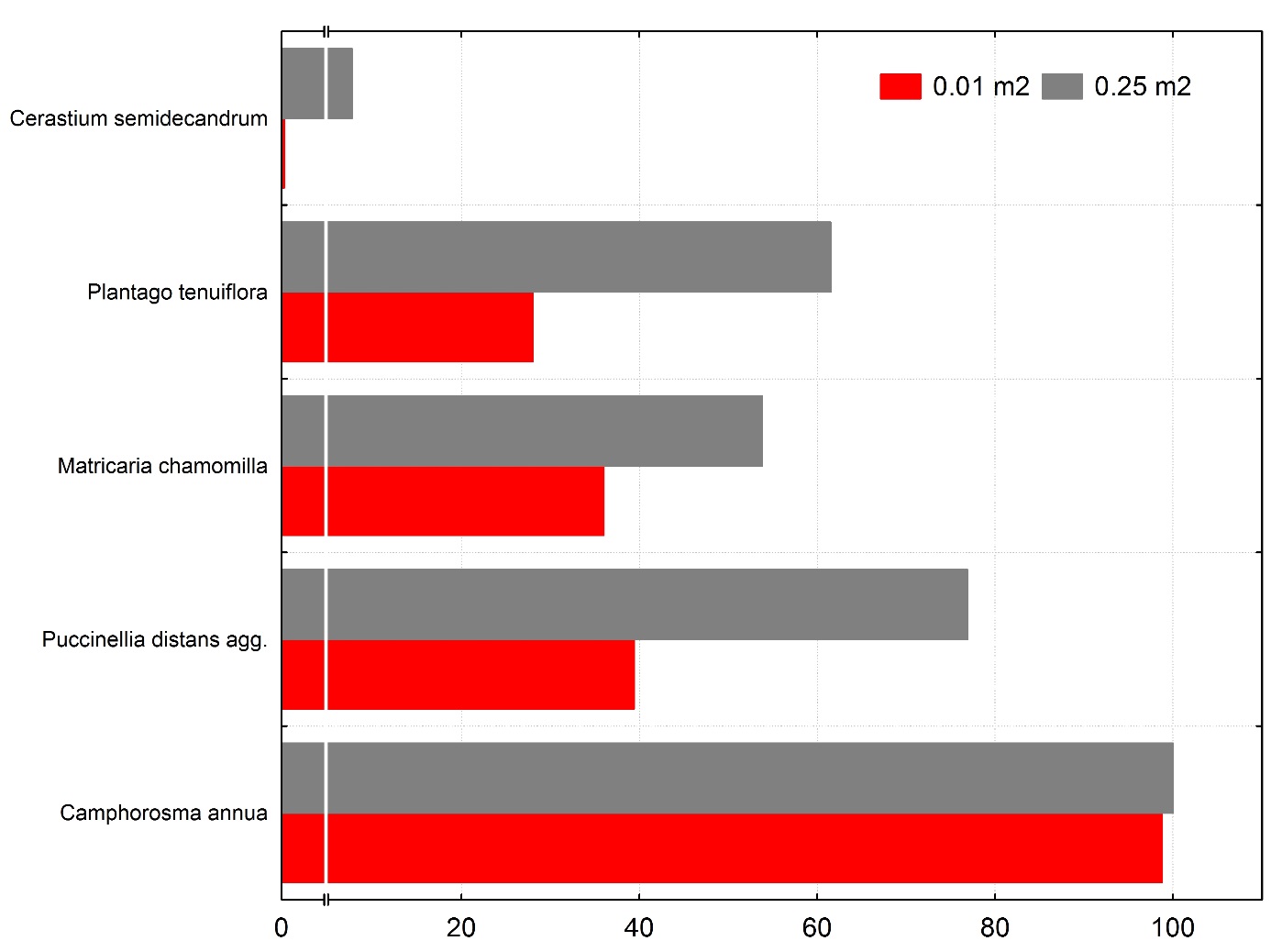
**

**Table S3.** Percentage frequency of all recorded vascular species in vegetation of *Puccinellietum limosae* (indicated by *Tripolium pannonicum* subsp. *pannonicum*) in different scales (0.01 m^2^ and 0.25 m^2^) surveyed in Slovakia (Podunajská nížina) and in Hungary (Kiskunság). All species were classified to characteristic species, including indicator species (first line, **bold**), accompanying halophytic species (second line, *italics*) and generalists indicating degradation (third line).

| *Tripolium pannonicum* plots | **Hungary** | | **Slovakia** | |
| --- | --- | --- | --- | --- |
|  | 250 subplots | 10 plots | 849 subplots | 34 plots |
|  | 0.01 m^2^  Percentage frequency | 0.25 m^2^  Percentage frequency | 0.01 m^2^  Percentage frequency | 0.25 m^2^  Percentage frequency |
| **Tripolium pannonicum** | **99.6** | 100.0 | 71.4 | 100.0 |
| **Puccinellia distans agg.** | **89.6** | 90.0 | 34.5 | 67.6 |
| **Plantago maritima** | **14.0** | 50.0 | 11.4 | 17.6 |
| *Juncus compressus* | 3.2 | 20.0 | 0.7 | 5.9 |
| *Bolboschoenus maritimus* | 1.2 | 10.0 | 0.4 | 5.9 |
| *Salsola soda* | 1.2 | 10.0 | 0.0 | 0.0 |
| *Festuca pseudovina* | 0.0 | 0.0 | 15.2 | 29.4 |
| *Artemisia santonicum* | 0.0 | 0.0 | 8.0 | 29.4 |
| *Juncus gerardii* | 0.0 | 0.0 | 5.2 | 5.9 |
| *Scorzonera cana* | 0.0 | 0.0 | 5.2 | 26.5 |
| *Atriplex littoralis* | 0.0 | 0.0 | 4.2 | 11.8 |
| *Limonium gmelinii* | 0.0 | 0.0 | 2.8 | 5.9 |
| *Camphorosma annua* | 0.0 | 0.0 | 1.8 | 2.9 |
| *Bupleurum tenuissimum* | 0.0 | 0.0 | 0.6 | 5.9 |
| *Triglochin maritima* | 0.0 | 0.0 | 0.5 | 2.9 |
| *Heleochloa schoenoides* | 0.0 | 0.0 | 0.2 | 2.9 |
| Elymus repens | 0.0 | 0.0 | 26.0 | 50.0 |
| Poa angustifolia | 0.0 | 0.0 | 15.1 | 32.4 |
| Agrostis stolonifera | 0.0 | 0.0 | 9.9 | 17.6 |
| Inula britannica | 0.0 | 0.0 | 8.7 | 20.6 |
| Lotus tenuis | 0.0 | 0.0 | 3.7 | 11.8 |
| Achillea millefolium | 0.0 | 0.0 | 3.2 | 8.8 |

* Other generalists, their percentage frequency on the scale of 0.01 m^2^ was lower than 3% in Slovakia, sorted by frequency value: Bromus hordeaceus, Carex stenophylla, Trifolium fragiferum, Polygonum aviculare, Lolium perenne, Festuca rupicola, Atriplex prostrata, Eryngium campestre, Arrhenatherum elatius, Cynodon dactylon, Schoenoplectus tabernaemontani, Juncus articulatus, Phragmites australis, Senecio erraticus, Vicia sativa , Festuca arundinacea, Sonchus arvensis, Centaurea jacea ssp. angustifolia, Centaurium pulchellum, Cerastium pumilum agg., Eleocharis uniglumis, Epilobium tetragonum, Hordeum geniculatum, Plantago media, Taraxacum sect. Ruderalia.

**Fig. S3.** Horizontal bar plots of percentage frequency of species recorded in two different scales (0,01 m^2^ – red, 0,25 m^2^ – grey) on reference plots of Hungary in vegetation of *Puccinellietum limosae.* On Axis X the scale break is marked at 5%, which is the limit for defining the characteristic species of particular vegetation type.


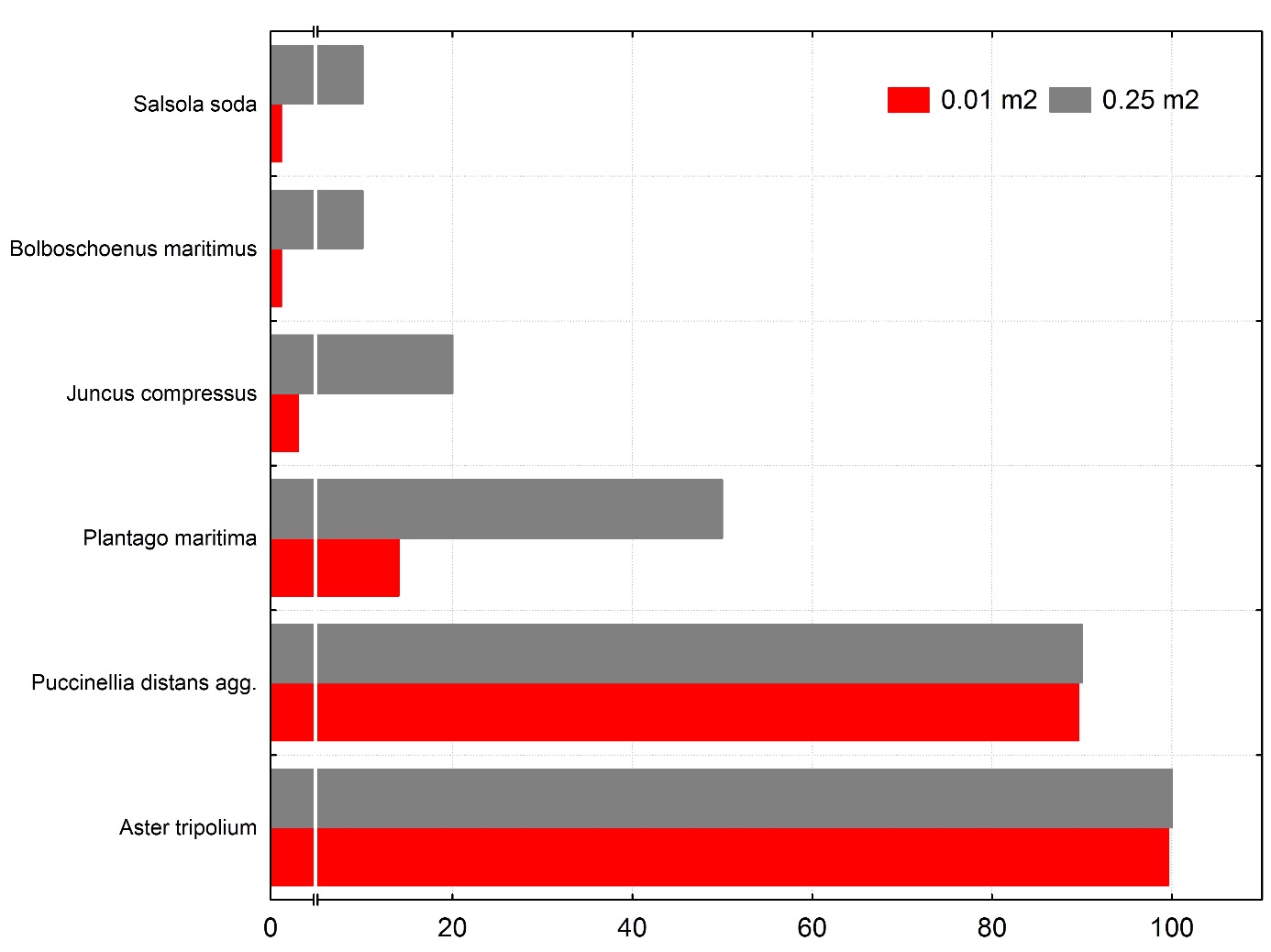

Supplement: Supplementary file 1 [file plants-10-00530-s001.zip › plants-1106238supplementary/S1_methods add revised.docx]
