# Supplementary material for: Current Condition of Pannonic Salt Steppes at Their Distribution Limit: What Do Indicator Species Reveal about Habitat Quality?
Source: Plants (Basel). 2021 Mar 11;10(3):530. doi: 10.3390/plants10030530 (PMC7999981; doi:10.3390/plants10030530)
Supplement: Supplementary file 1 [file plants-10-00530-s001.zip › plants-1106238supplementary/S2_photo of plots revised.pdf]

Supplementary material for research article

Current condition of Pannonic salt steppes at their distribution limit: what do indicator species reveal about habitat quality?

Zuzana Dítě, Róbert Šuvada, Tibor Tóth, Pavol Eliáš, Vladimír Píš & Daniel Dítě

Submitted to PLANTS journal

S2_Photographs of vegetation plots with three flagship species of salt steppes taken by the authors during field sampling

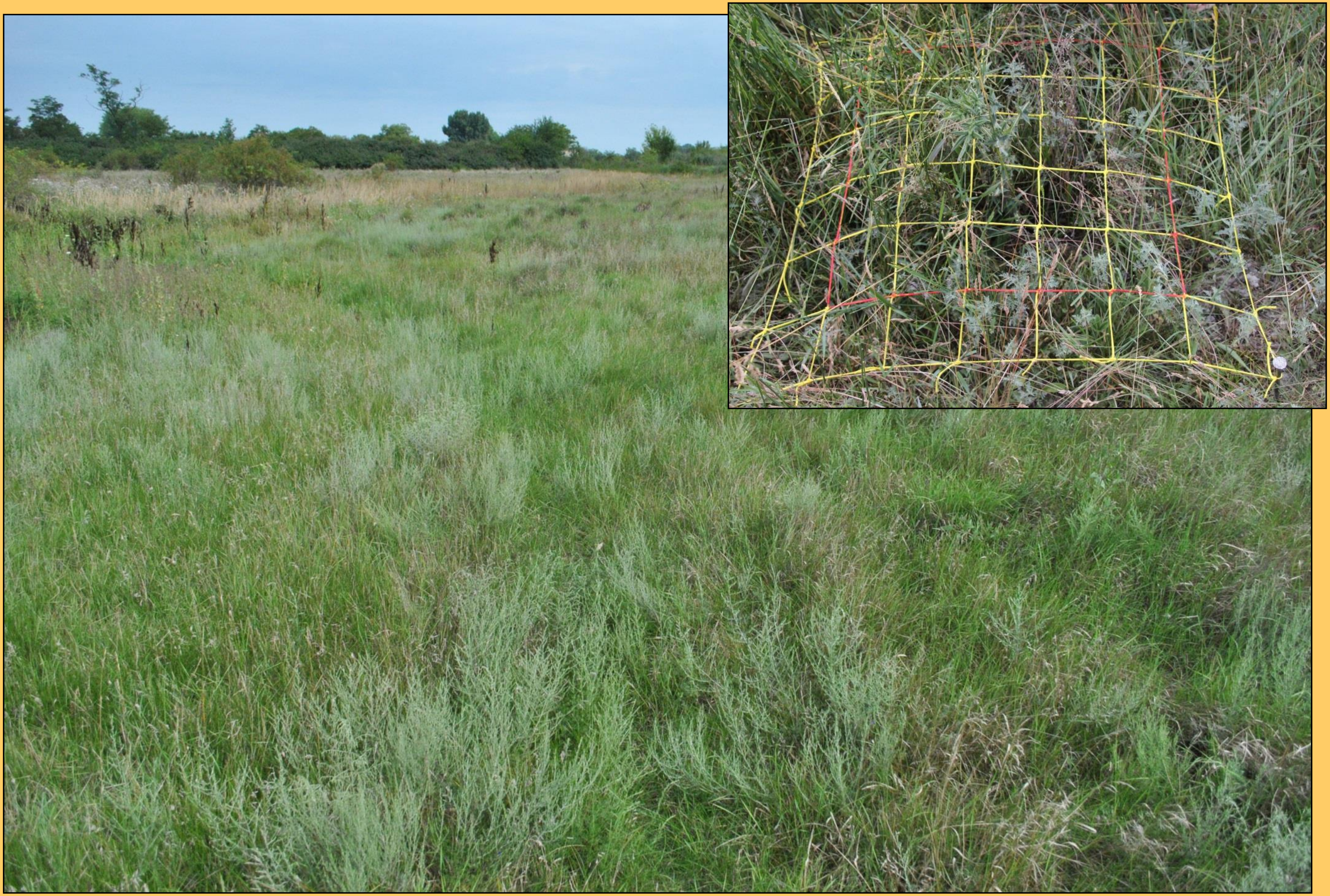

Degraded *Artemisia* steppe, vegetation plot with indicator species *Artemisia santonicum* subsp. *patens*, recorded in Slovakia, site no. 7 in Supplementary material S3, group of naturalness 4.

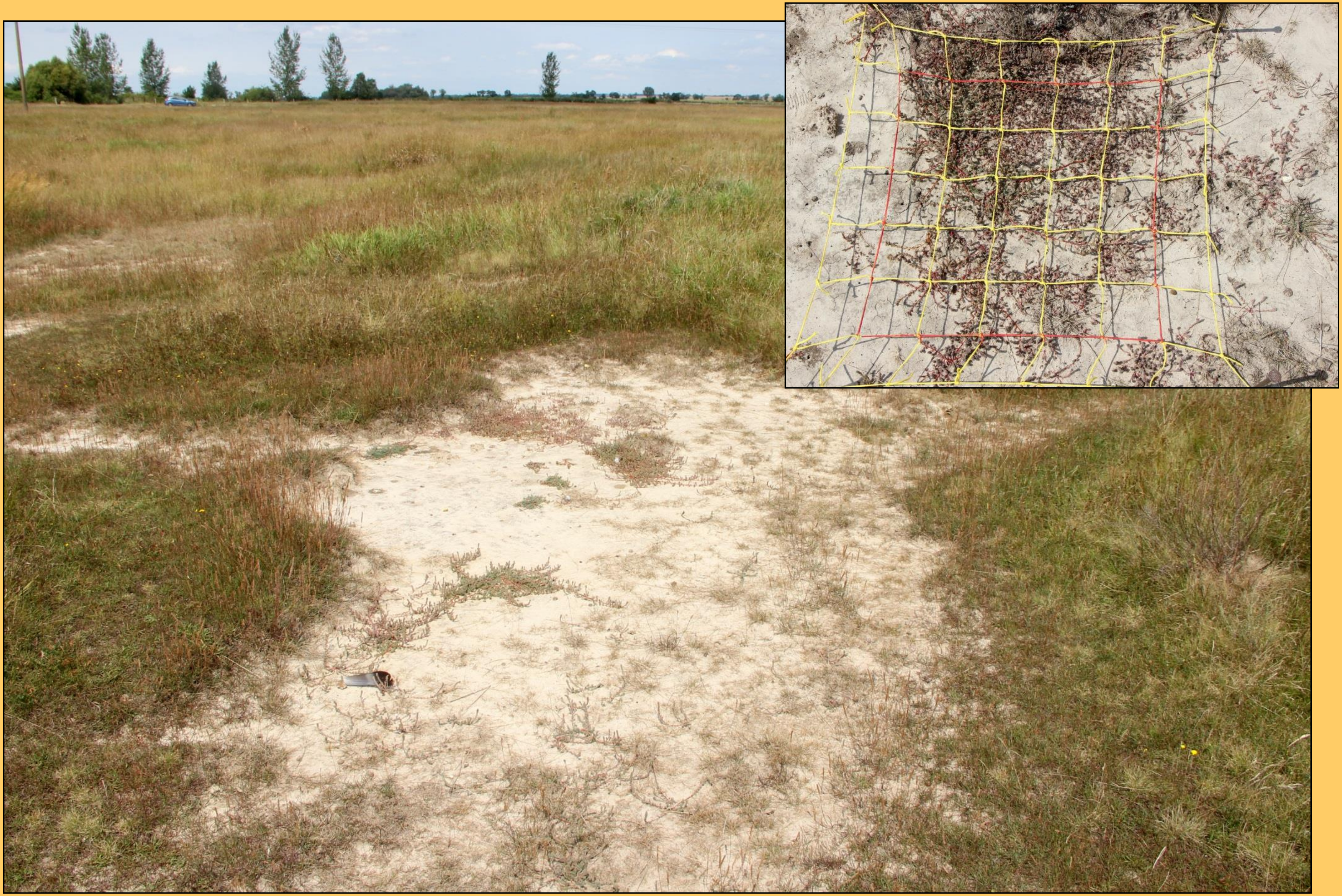

Barren spots of alkali soils occuppied by therophyte stands of *Camphorosma annua*, plot recorded in Slovakia, site no. 10 in Supplementary material S3, group of naturalness: 1.

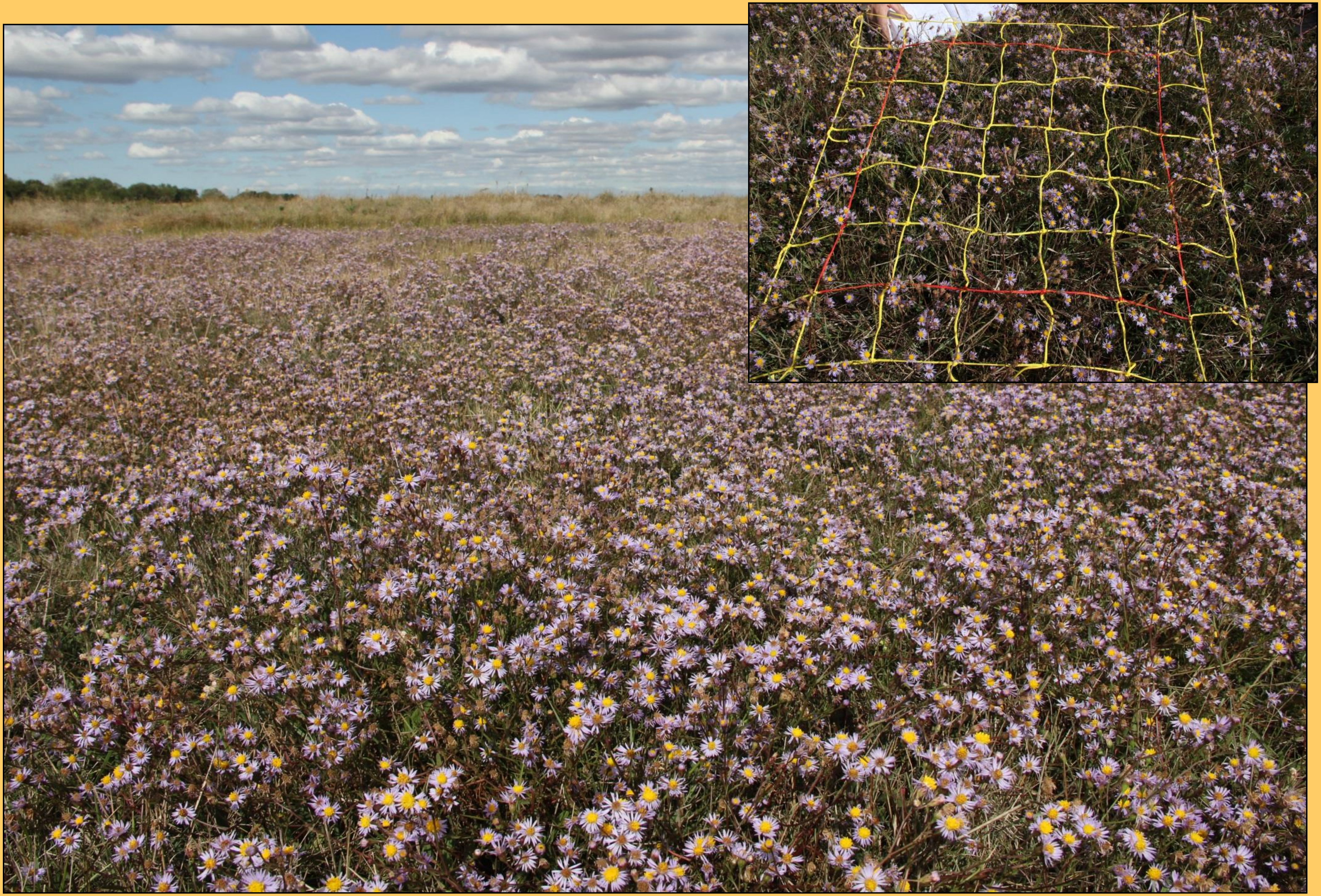

Flat depressions on saline soils with degraded stands of *Tripolium pannonicum* subsp. *pannonicum*, plot recorded in Slovakia, site no. 23 in Supplementary material S3, group of naturalness 3.

Reference vegetation of preserved Pannonic salt steppes with characteristic zonation in Hungary

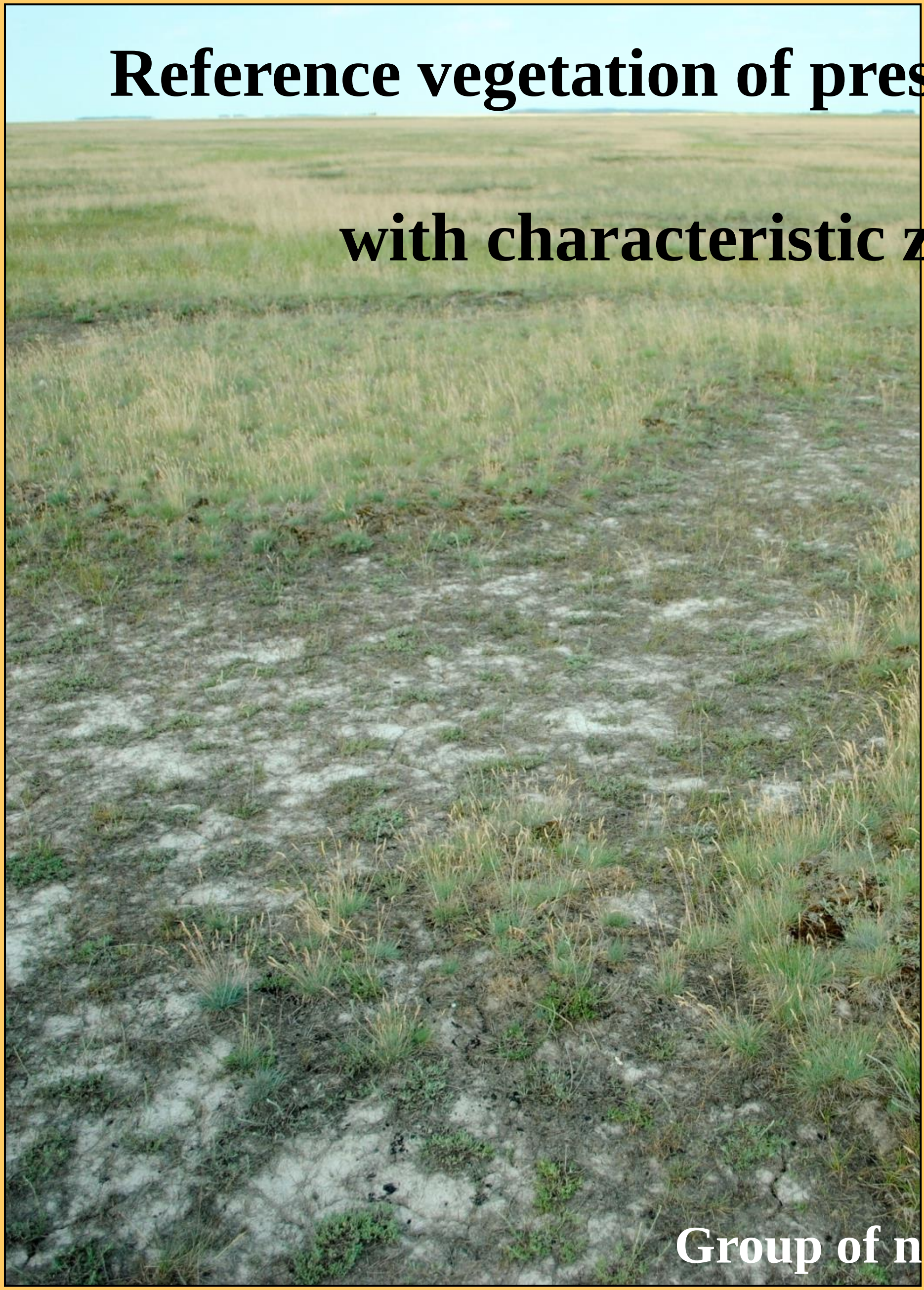

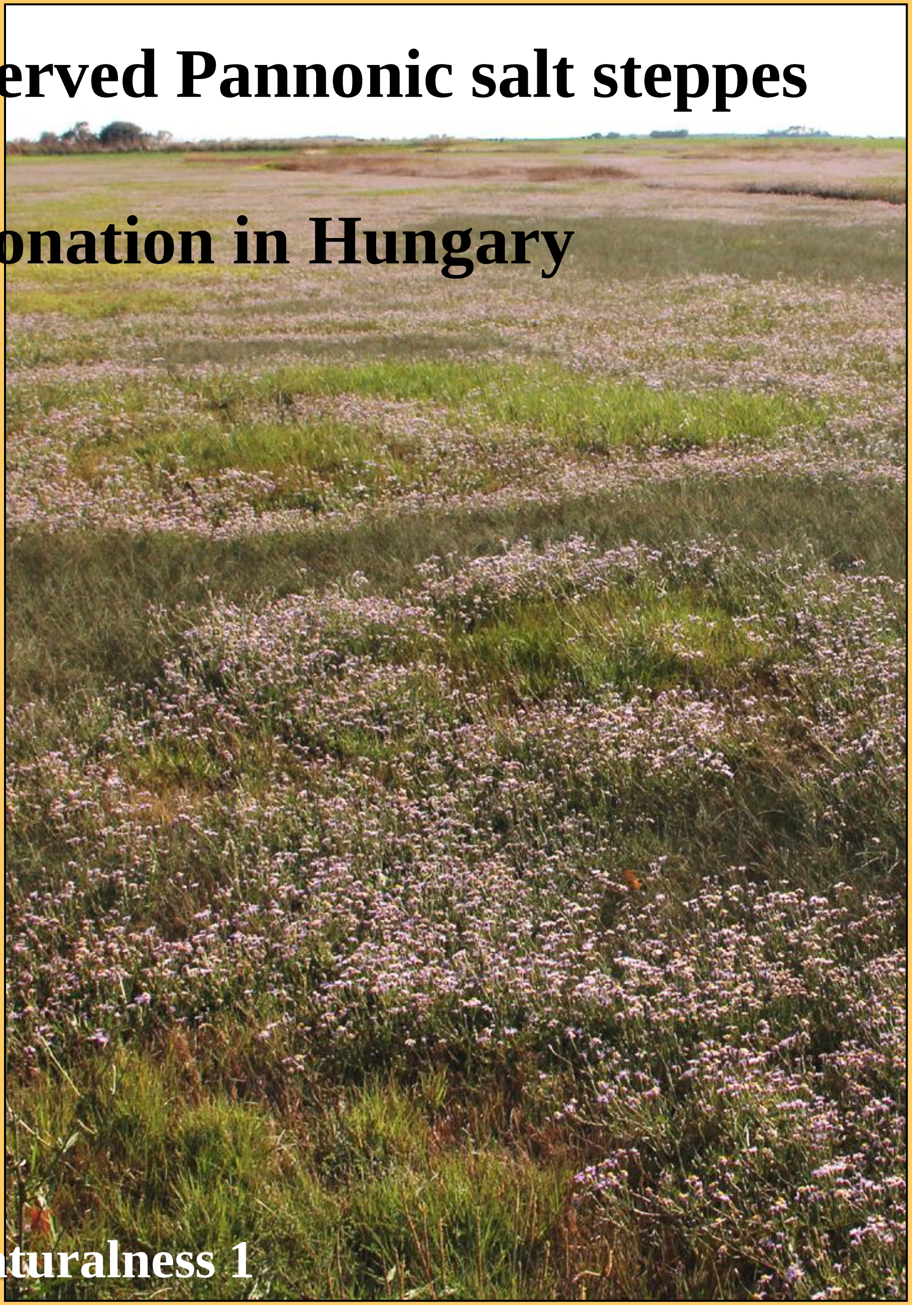

Group of naturalness 1

Mosaic of alkali habitats: *Artemisia* steppes and barren spots with *Camphorosma annua* in Hortobágy.

Flat saline depressions with stands of *Tripolium pannonicum* subsp. *pannonicum* in Kiskunság.
